# Trypanocidal Effects of Polyprenylated Benzophenone Enriched Brazilian Red Propolis Extract and Its Isolated Constituent

**DOI:** 10.1002/cbdv.202502682

**Published:** 2026-01-18

**Authors:** Nicoli Dias Oliveira, Mariana Cintra Pagotti, Lucas Antonio de Lima Paula, Mariana Zaramello Paixão, Daiane Albino dos Santos, Renata Faleiros de Freitas, Sérgio Ricardo Ambrósio, Rodrigo Cassio Sola Veneziani, Jairo Kenupp Bastos, Lizandra Guidi Magalhães

**Affiliations:** ^1^ Center For Research in Sciences and Technology University of Franca. Avenida Dr. Armando Salles of Oliveira Franca Brazil; ^2^ School of Pharmaceutical Sciences of Ribeirão Preto University of São Paulo. Av. Do Café Ribeirão Preto Brazil

**Keywords:** *American trypanosomiasis*, Brazilian red propolis, guttiferone E/xanthochymol, oblongifolin B, *Trypanosoma cruzi*

## Abstract

Chagas disease, a neglected tropical disease, affects millions of individuals in developing countries. Due to the limitations of current therapeutic options, the search for new bioactive compounds is crucial. Brazilian red propolis, a resinous bee product from northeastern Brazil, is recognized for its diverse chemical constituents and biological, including antiparasitic, activities. This study evaluated the in vitro activity of a polyprenylated benzophenone‐enriched Brazilian red propolis extract (**SEBz**) and its constituents, the mixture of isomers guttiferone E and xanthochymol (**GUT/XAN**), and oblongifolin B (**OBL**), against *Trypanosoma cruzi* (Y strain) trypomastigote and amastigote forms. Cytotoxicity and hemolysis assays assess safety. **SEBz** exhibited antiparasitic activity, with 50% effective concentration (EC_50_) values of 17.97 µg mL^−1^ (trypomastigotes) and 6.83 µg mL^−1^ (amastigotes) after 48 h, while demonstrating moderate cytotoxicity on the C2C12 myoblast cell line (50% cytotoxicity concentration [CC_50_] 42.80 µg mL^−1^ at 48 h). Both **GUT/XAN** and **OBL** showed trypanocidal effects (EC_50_ ≤ 5 µM against trypomastigotes; **GUT/XAN** EC_50_ 7.91 µM on amastigotes), with moderate cytotoxicity (CC_50_ < 20 µM). Neither the extract nor the compounds induced significant hemolysis. Ultrastructural analysis of treated parasites revealed nuclear deformation and vacuolation. These findings support the potential of Brazilian red propolis benzophenones as promising leads for Chagas disease drug development.

## Graphical abstract

In vitro trypanocidal activity of a polyprenylated benzophenone‐enriched Brazilian red propolis extract (SEBz) and its major constituents, guttiferone E/xanthochymol (GUT/XAN) and oblongifolin B (OBL), exhibit in vitro trypanocidal activity against *Trypanosoma cruzi*, inducing ultrastructural damage with moderate cytotoxicity and no significant hemolysis, highlighting their potential as leads for Chagas disease drug development.

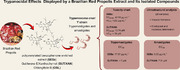

## 1 Introduction

*Trypanosoma cruzi*, a hemoflagellate protozoan, is known for being the etiologic agent of Chagas disease (American trypanosomiasis), a neglected tropical disease throughout Latin America, with over 7 million infected individuals globally and around 30 000 new cases annually [1, 2]. The chronic phase manifests with severe cardiac, digestive, and neurological complications, often resulting in fatal outcomes [1].

Current treatment strategies for Chagas disease rely primarily on two nitroheterocyclic drugs: benznidazole (BNZ) and nifurtimox (NFX). These agents are most effective during the acute phase of the disease, when parasite replication is high, yet their efficacy decreases in the chronic phase. Furthermore, both drugs are associated with significant adverse effects, leading to treatment discontinuation in a notable proportion of patients [3]. From this perspective, researchers are intensifying their efforts to develop different approaches to treat American trypanosomiasis, highlighting the studies with natural products, since they are substantial sources of new biologically active compounds [4, 5, 6, 7].

Propolis, a resinous bee product, has been used in alternative medicine worldwide since ancient times as a natural antibiotic. It has recently become the focus of scientific research due to its broad range of properties and therapeutic actions, including antimicrobial, anticancer, anti‐inflammatory, antiviral, antioxidant, anesthetic, healing, hepatoprotective, and hematoprotective effects [8, 9, 10]. Honey bees (*Apis mellifera)* make propolis from a mixture of plant exudates, young botanical tissues and buds, pollen, wax, with their own saliva and enzymes; features that confer an intrinsic relation between botanical sources and chemical composition of propolis [11]. In honeycombs, propolis displays both structural and protective properties, being used to repair wall cracks, maintain appropriate humidity and heat levels, and also contributing to keeping a pathogen‐free environment, as well as keeping invaders away [10]. The wide biodiversity in Brazil is now responsible for 13 different types of propolis, differing in color and in their physicochemical properties, which are closely influenced by the geographic origin and seasonal variations of the plant sources involved in their production [12, 13].

**FIGURE 1:**
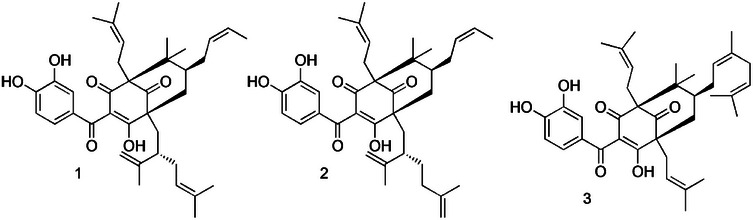
Chemical structures of compounds identified in polyprenylated benzophenone‐enriched Brazilian red propolis extract: the mixture of guttiferone E (**1**), xanthochymol (**2**)—**GUT/XAN**, and oblongifolin B—**OBL** (**3**).

Red propolis, found on the northeastern side of Brazil, has as its botanical source two distinct trees, *Dalbergia ecastaphyllum* (L.) Taub. and *Symphonia globulifera* L.f. (Clusiaceae) [14]. While *D. ecastophyllum* is mainly responsible for the phenolic compounds, such as liquiritigenin, isoliquiritigenin, formononetin, vestitol, neovestitol, medicarpin, and 7‐O‐neovestitol; *S. globulifera* appears as the source of guttiferone E (**GUT**), xanthochymol (**XAN**), and oblongifolin B (**OBL**), both polyprenylated benzophenones [14, 15]. Although red propolis extract demonstrates promising biological activities, including anti‐inflammatory [16], antimicrobial [17], and antiproliferative [18] properties, some studies carried out with Brazilian red propolis extracts showed antiprotozoal activity against *Leishmania* sp. [19, 20, 21, 22], *Trypanosoma* sp. [19, 23, 24, 25, 26, 27], and *Trichomonas vaginalis* [28]. Therefore, this work was focused on investigating the in vitro biological effects of a polyprenylated benzophenone‐enriched Brazilian red propolis extract (**SEBz**), as well as its compounds, the mixture of double bond isomers **GUT/XAN** and **OBL** (Figure 1) against trypomastigotes and amastigotes, *T. cruzi* (Y strain), assessing the ultrastructural damages induced in parasites, besides evaluating the cytotoxic and hemolytic properties.

## 2 Results and Discussion

The standardization of enriched extracts is a crucial step in natural product research, particularly to ensure the reproducibility of biological results, safety, and pharmacological efficacy. The chemical composition of extracts, such as propolis, can vary significantly depending on botanical and geographical origin, seasonality, extraction methods, and storage conditions [21, 29, 30]. Without standardization, results from different studies become difficult to compare, hindering progress in the development of phytopharmaceuticals or clinical formulations. Standardization allows for the identification and quantification of bioactive chemical markers, contributing to quality control and accurate therapeutic dosing [30]. In this study, experiments were conducted with **SEBz**, a polyprenylated benzophenone‐enriched extract, obtained previously [31], and which contains benzophenones **GUT/XAN** and **OBL** at 16.68% and 42.25% of the total extract content, respectively [31]. Also, in this work, the effects of isolated compounds, **GUT/XAN** and **OBL**, were evaluated.

**FIGURE 2:**
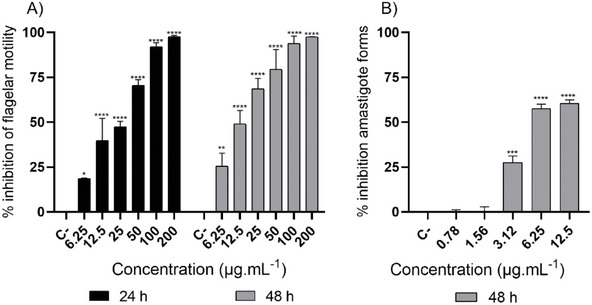
Effects of **SEBz** against trypomastigotes and amastigotes of *T. cruzi* (Y strain). Percentage of inhibition of flagellar motility in vitro against the trypomastigote forms after incubation with **SEBz** at 24 and 48 h (A). Percentage of inhibition against the amastigote forms after 48 h of incubation with **SEBz** (B). Asterisk denotes statistical difference compared to the negative control (parasites cultivated in medium containing 0.1% DMSO) * *p* < 0.05, ** *p* < 0.01, *** *p* < 0.001, and **** *p* < 0.0001.

For the screening of trypanocidal activity, the quantification of live *T. cruzi* trypomastigotes was performed using light microscopy and direct counting in a Neubauer chamber, considered the gold standard for phenotypic assays [32]. Although motility‐based evaluation may not fully capture viability, since some effects can be reversible, this method remains widely used in *Trypanosoma* spp. screening due to its simplicity and reliability [32]. Additionally, the use of C2C12 myoblasts as host cells for the amastigote assay can be justified by their robustness and susceptibility to *T. cruzi* infection [33]. Nevertheless, complementary approaches using human‐derived cell lines are warranted to validate these results and enhance translational relevance. Against trypomastigote forms, our results demonstrated significant trypanocidal activity of **SEBz** at all evaluated concentrations. **SEBz** exhibited a flagellar motility inhibition of 70.59% and 47.46% at concentrations of 50 and 25 µg mL^−1^, respectively, at 24 h (Figure 2A), and a flagellar motility inhibition of 68.71% and 49.09% at concentrations of 25 and 12.5 µg mL^−1^, respectively, at 48 h (Figure 2A). The 50% effective concentration (EC_50_) values for 24 and 48 h were 21.64 and 17.97 µg mL^−1^, respectively (Table 1). Against amastigote forms, **SEBz** exhibited significant activity at concentrations ranging from 12.5 to 3.12 µg mL^−1^, showing a percentage reduction of amastigote forms of 60.55% at the concentration of 12.5 µg mL^−1^ after 48 h of incubation (Figure 2B) and an EC_50_ value of 6.83 µg mL^−1^ (Table 1). The antiparasitic activity of extracts can be classified against protozoa as highly active (EC_50_ < 10 µg mL^−1^), active (EC_50_ between 10 and 50 µg mL^−1^), moderately active (EC_50_ between 50 and 100 µg mL^−1^), and non‐active (EC_50_ >100 µg mL^−1^) [34]. In this sense, **SEBz** was considered highly active against the amastigote forms of *T. cruzi* (Y strain).

**TABLE 1:** In vitro cytotoxicity, hemolytic, and trypanosomal activities.

	CC_50_		EC_50_			SI			HC_50_
	C_2_C_12_		Trypomastigote		Amastigote		Trypomastigote		Amastigote
	24 h	48 h	24 h	48 h	48 h	24 h	48 h	48 h	3 h
**SEBz**	51.32 (45.67–57.17)	42.80 (28.07–62.35)	21.64 (17.55–26.47)	17.97 (12.75–24.75)	6.83 (4.99–10.24)	2.37	2.38	6.26	>200
**GUT/XAN**	18.61 (12.54–26.65)	12.70 (11.67–13.87)	7.85 (6.25–9.78)	5.15 (4.13–6.34)	7.91 (7.04–8.96)	2.37	2.46	0.62	>50
**OBL**	12.64 (9.27–17.07)	11.53 (10.99–12.07)	6.92 (5.71–8.33)	4.74 (3.77–5.88)	11.63 (9.24–13.94)	1.82	2.43	1.00	>50
**BNZ**	59.36 (41.69–127.40)	47.76 (29.49–115.80)	8.33 (6.05–11.41)	3.59 (2.94–4.30)	5.49 (4.53–6.47)	7.12	13.30	8.70	>50

**CC_50_ –** cytotoxic concentration to 50% of the cells. **EC_50_ –** effective concentration to 50% of the parasites**. SI** – selectivity index**. HC_50_ –** hemolytic concentration to 50% of red cells**. C2C12 –** myoblast cell line (ATCC: CRL‐1772). **SEBz** – polyprenylated benzophenone enriched Brazilian red propolis extract t (µg mL^−1^). **GUT/XAN** – guttiferone E/xanthochymol (µM); **OBL** – oblongifolin B (µM), and **BNZ** – benznidazole (µM). Concentration between parentheses represents a 95% confidence interval.

Although interest in the effects of propolis extracts on parasitic diseases has increased, studies evaluating the efficacy of Brazilian red propolis against *T. cruzi*, particularly the Y strain, remain limited [19, 23, 24, 25, 26]. Previous investigations have shown that red propolis extracts display antiparasitic activity against *T. cruzi*, although most focused on early parasite stages or used non‐enriched extracts [19, 24]. Ethanolic and hydroethanolic red propolis extracts from different Brazilian regions exhibited moderate to high activity at concentrations ranging from 75 to 500 µg mL^−^^1^ against epimastigote forms of *T. cruzi*, which correspond to the replicative stage in the invertebrate host [19, 24]. Studies have also shown notable activity of red propolis extracts against *Trypanosoma brucei*, the causative agent of African trypanosomiasis [23, 26, 27]. Extracts from both Nigeria and Brazil, particularly those rich in phenolic compounds, displayed moderate to high activity, and the observed efficacy was correlated with total phenolic content [23, 26, 27]. However, the specific contribution of benzophenone derivatives was not assessed in those studies.

**FIGURE 3:**
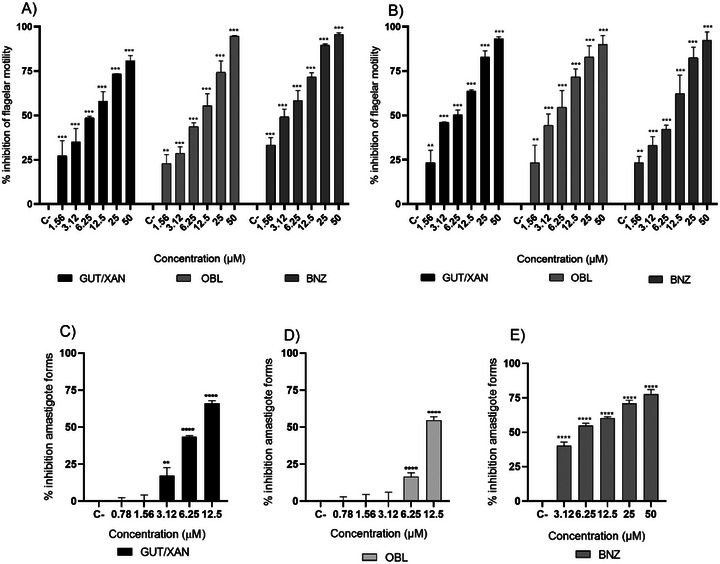
Effects of compounds **GUT/XAN**, **OBL**, and BNZ against trypomastigotes and amastigotes of *T. cruzi* (Y strain). Percentage of inhibition of flagellar motility in vitro against the trypomastigote forms after incubation with the compounds at 24 (A) and 48 h (B). Percentage of inhibition against the amastigote forms after 48 h of incubation with **GUT/XAN** (C), **OBL** (D), and BNZ (E). Asterisk denotes statistical difference compared to the negative control (parasites cultivated in medium containing 0.1% DMSO) ^*^ *p* < 0.05, ^**^ *p* < 0.01, ^***^ *p* < 0.001, and ^****^ *p* < 0.0001.

Given the variation in the chemical composition of propolis depending on its botanical and geographical origin, further phytochemical characterization is essential to identify the active constituents responsible for the observed activity [21, 29]. In this context, the compounds **GUT/XAN** and **OBL** also exhibited significant trypanocidal activity against trypomastigote forms at all evaluated concentrations, similar to the standard drug BNZ. At 24 h, the inhibition of flagellar motility was 74.32%, 73.32%, and 82.43% at a concentration of 25 µM for **GUT/XAN, OBL**, and BNZ, respectively (Figure 2A). At 48 h, the inhibition of flagellar motility was 82.79% for **GUT/XAN** and **OBL**, and 89.49% for BNZ at a concentration of 25 µM (Figure 3B). The EC_50_ values at 24 h were 7.85, 6.92, and 8.33 µM (4.74, 4.18, and 2.17 µg mL^−1^) for **GUT/XAN**, **OBL,** and BNZ, respectively, and at 48 h, the EC_50_ values were 5.15, 4.74, and 3.59 µM (3.11, 2.86, and 0.93 µg mL^−1^), respectively (Table 1). Also, significant activity of **GUT/XAN** was observed, with a percentage reduction of amastigote forms of 66.07% at the concentration of 12.5 µM after 48 h of incubation (Figure 3C). **OBL** showed significant activity only at concentrations of 12.5 and 6.25 µM, with a percentage reduction of amastigote forms of 54.67% at the concentration of 12.5 µM, also after 48 h of incubation (Figure 3D). BNZ showed significant activity at all tested concentrations, with a percentage reduction of amastigote forms of 77.73% at the concentration of 50 µM (Figure 3E). The compound **GUT/XAN** exhibited the lowest EC_50_ value (7.91 µM; 4.78 µg mL^−^^1^), which was comparable to that of BNZ (EC_50_ = 5.49 µM; 1.43 µg mL^−^^1^). In contrast, **OBL** showed the highest EC_50_ value (11.63 µM; 7.01 µg mL^−^^1^) (Table 1). The superior activity of **SEBz** and its isolated constituents (**GUT/XAN** and **OBL**) validates the strategy of targeting these specific compounds, which are the main active agents, for enhanced efficacy and standardization. While crude extracts are complex and variable, the enriched profile provides a more potent and reproducible antiparasitic effect, with the isolated benzophenones demonstrating high micromolar potency against the clinically relevant form, offering novel insights into the mechanism of action through observed ultrastructural damage [19, 24, 26].

The trypanocidal and leishmanicidal potential of prenylated benzophenones, particularly guttiferone A, has been widely documented [35, 36, 37]. Although these compounds share the same chemical backbone, subtle variations in the number and position of prenyl and hydroxyl groups attached to the benzophenone core can lead to distinct biological activities [38]. Guttiferone A has shown potent effects against protozoan parasites such as *Plasmodium falciparum*, likely through interference with mitochondrial redox balance [35]. Further studies revealed that guttiferone A and the related benzophenone nemorosone inhibit mitochondrial respiratory complexes II and III, disrupting parasite bioenergetics and redox homeostasis [36]. Efforts to enhance the bioactivity and selectivity of guttiferone A have resulted in several generations of semisynthetic analogues with improved antiparasitic potency against *T. brucei*, *Leishmania donovani*, and *P. falciparum* [39, 40]. These findings reinforce that targeted structural modifications of the benzophenone scaffold can optimize both pharmacological and physicochemical properties. Overall, our findings are consistent with the literature, suggesting that the biological activity of **SEBz** is likely associated with the presence of benzophenone derivatives such as **GUT/XAN** and **OBL**.

**FIGURE 4:**
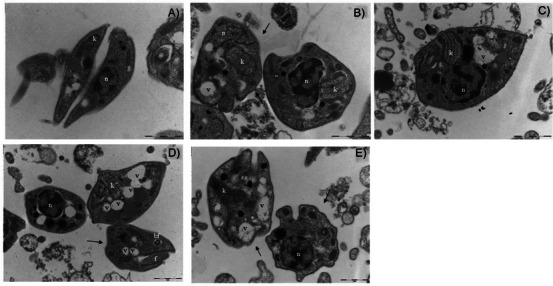
Ultrastructural changes in *T. cruzi* trypomastigotes after 48 h incubation with **SEBz** and isolated compounds. Images acquired using transmission electron microscopy. (A) Negative control (parasites cultivated in medium containing 0.1% DMSO), (B) Benznidazole (BNZ), (C) **SEBz**, (D) **GUT/XAN**, and (E) **OBL**. Plasma membrane (arrow); Nucleus (N); Kinetoplast (k); Flagellar pocket (bf); Vacuole (v).

To investigate the morphological effects of **SEBz** and its isolated constituents on *T. cruzi*, transmission electron microscopy (TEM) was performed on trypomastigote forms incubated for 48 h with the extract and isolated compounds at their respective EC_50_ concentrations. Control parasites exhibited preserved nuclear morphology and a kinetoplast with normal size and organization (Figure 4A). In contrast, parasites exposed to **SEBz** or its isolated compounds displayed evident morphological disruptions, including nuclear deformation and swelling of the kinetoplast. Notably, the presence of cytoplasmic vacuoles was more prominent in parasites incubated with **GUT/XAN** and **OBL** (Figure 4D,E). Alterations in the integrity of the plasma membrane were also observed in parasites incubated with **GUT/XAN, OBL** (Figure 4D,E), and BNZ (Figure 4B). Furthermore, all groups exposed to the extract and compounds, including those exposed to the reference drug BNZ, demonstrated a characteristic rounding of the parasite body, a feature absent in the control group. These findings suggest that both **SEBz** and its bioactive constituents exert marked ultrastructural effects on *T. cruzi* trypomastigotes, potentially compromising parasite viability.

One of the most striking morphological alterations observed was the swelling of the kinetoplast in all experimental groups, with the most pronounced changes detected in parasites treated with BNZ and **SEBz**. The kinetoplast is a distinctive and essential mitochondrial DNA‐containing structure in kinetoplastid parasites, playing a key role in mitochondrial metabolism, cell division, and parasite infectivity [41]. Structural damage or swelling of the kinetoplast has been associated with mitochondrial membrane depolarization and impaired energy metabolism, ultimately leading to parasite death [42]. Polyprenylated benzophenone compounds are known for their pro‐oxidant activity at high concentrations and their ability to target mitochondrial structures [43]. Consistent with these prior observations, the effects observed may be related to redox imbalance and mitochondrial disruption induced by **SEBz** and its active constituents, contributing to the structural disintegration observed in trypomastigotes. Nevertheless, further studies integrating biochemical assays with transcriptomic and proteomic analyses will be essential to elucidate the precise molecular targets and pathways affected by red propolis‐derived compounds.

The superior activity of our polyprenylated benzophenone‐enriched extract (**SEBz**) and its isolated constituents (**GUT/XAN** and **OBL**) validates the strategy of targeting these specific compounds, which are the main active agents, for enhanced efficacy and standardization. While crude extracts are complex and variable, the enriched profile provides a more potent and reproducible antiparasitic effect, with the isolated benzophenones demonstrating high micromolar potency against the clinically relevant amastigote form, offering novel insights into the mechanism of action through observed ultrastructural damage.

The evaluation of toxicity represents a fundamental step in the screening of natural products for potential chemotherapeutic applications [19, 31]. In the present study, the cytotoxic effects of **SEBz** and the compounds were assessed using the C2C12 mouse myoblast cell line. As shown in Table 1, **SEBz** exhibited lower cytotoxicity compared to the isolated compounds, with 50% cytotoxicity concentration (CC_50_) values of 51.32 µg mL^−1^ at 24 h and 42.80 µg mL^−1^ at 48 h. Despite this, the selectivity index (SI) indicated that **SEBz** was approximately 2‐fold more selective against the trypomastigote forms and 6‐fold more selective against the amastigote forms of the parasite, when compared to its effect on mammalian cells. In contrast, the isolated compounds **GUT/XAN** and **OBL** demonstrated higher cytotoxicity, with CC_50_ values of 18.61 and 12.64 µM (equivalent to 11.23 and 7.62 µg mL^−1^) at 24 h, and 12.70 and 11.53 µM (7.66 and 6.96 µg mL^−1^) at 48 h, respectively. These compounds were approximately 2.5 times more effective against trypomastigote forms; however, their selectivity against amastigotes was limited, with SI values ranging from 1.11 to 1.82, indicating a narrow therapeutic window (Table 1). For comparison, the reference drug BNZ presented CC_50_ values greater than 50 µM at 24 h, and demonstrated higher selectivity, with SI values of approximately 13 and 9 against trypomastigote and amastigote forms, respectively, at 48 h. Additionally, no hemolysis was observed under the experimental conditions evaluated, indicating a favorable safety profile regarding erythrocyte integrity.

Previous studies corroborate the moderate to high cytotoxic potential of red propolis and its constituents. In non‐tumorigenic breast epithelial cells (MCF‐10A), **SEBz, GUT/XAN,** and **OBL** showed CC_50_ values of 19.58 µg mL^−1^, 18.23 µM, and 31.46  µM, respectively [31]. Similarly, hydroalcoholic extracts of red propolis from different Brazilian regions showed CC_50_ values ranging from 65 to 85 µg mL^−1^ in murine embryo fibroblasts (BALB/c 3T3) [44], supporting the notion that, despite their biological activity, red propolis extracts may exert significant cytotoxic effects on non‐target cells. Although the low in vitro SI values observed for **GUT/XAN** and **OBL** indicate a narrow therapeutic window, this finding does not preclude their potential as antiparasitic leads. When compared with other natural trypanocidal agents, the SI values obtained here fall within the lower range of those reported, which often exhibit modest selectivity (SI ≈ 2–4) prior to chemical optimization [45, 46]. In contrast, optimized derivatives or semisynthetic analogues generally achieve SI > 10, a threshold commonly regarded as indicative of a promising lead compound [46, 47]. Thus, the current data suggest that **SEBz** and its benzophenone constituents represent preliminary scaffolds requiring further refinement to enhance safety and selectivity. Complementary strategies may help address these limitations. For example, structure–activity relationship (SAR) studies could guide targeted modifications of prenyl or hydroxyl groups to attenuate cytotoxicity while maintaining antiparasitic potency, and formulation or delivery approaches, including encapsulation or nanoparticle systems, could limit host exposure while improving pharmacokinetics, thereby expanding the therapeutic window. Additionally, further cytotoxicity profiling in mammalian cells, combined with mechanistic assays, will help determine whether host‐cell damage occurs.

## 3 Conclusions

Given the results obtained, it can be concluded that **SEBz** exhibits potent in vitro trypanocidal activity against the amastigote forms of *Trypanosoma cruzi* (Y strain), with moderate cytotoxicity. Among the compounds evaluated, **GUT/XAN** and **OBL** also demonstrated activity against both amastigote and trypomastigote forms of *T. cruzi* (Y strain), though with lower selectivity relative to mammalian cells. These findings pave the way for further investigations aimed at elucidating the mechanisms of action of these compounds and other propolis‐derived extracts. Additionally, in vivo studies would be highly valuable to better understand the antiparasitic, cytotoxic, and immunomodulatory properties of Brazilian red propolis extract and/or its active constituents. Moreover, these studies will also contribute to expanding the potential medicinal applications of this type of bee product.

## 4 Experimental

### 4.1 Preparation of Extract and Isolation of Compounds

Red propolis was purchased from Cooperativa de Apicultores de Canavieiras in the city of Canavieiras (Bahia state, Brazil) in April 2018. The polyprenylated benzophenone‐enriched extract and its constituents, the mixture of **GUT/XAN** and **OBL**, were obtained previously as described by Pires et al. [31]. Chromatographic analyses of **SEBz** were performed using reverse phase‐high‐performance liquid chromatography‐photo diode array (RP‐HPLC‐PDA) with a Shim‐pack VP‐ODS column (250 mm × 4.6 mm i.d., 5 µm; Shimadzu, Kyoto, Japan, Cat# 228‐34937‐92). The flow rate was set at 1 mL/min, and ultraviolet detection was conducted at 250 nm. A mobile phase gradient system was employed, consisting of 0.1% acetic acid in water (solvent A) (Sigma–Aldrich, St. Louis, USA, Cat#33209) and acetonitrile (solvent B) (Sigma‐Aldrich, Cat#34967). The gradient ranged from 85% to 100% of solvent B over 20 min. The column temperature was maintained at 40°C, and the injected volume was 20.0 µL. The analytical equation for **GUT/XAN** was *y* = 0.003*x* – 0.040 (*r* ^2^ = 0.998; limit of detection [LOD] = 0.462 µg mL^−1^ and quantification [LOQ] = 1.400 µg mL^−1^; the recovery mean was 92.422%). As for **OBL**, the analytical equation was *y* = 0.001*x* ‐ 0.015 (r^2^ = 0.996; LOD = 5.057 µg mL^−1^ and LOQ = 15.323 µg mL^−1^; the recovery mean was 96.888%) [31].

The purity of the isolated compound **OBL** and the mixture of double bond isomers **GUT/XAN** were estimated to be greater than 98% through both HPLC and nuclear magnetic resonance (NMR) analysis [31]. The chemical structures of the compounds were determined using one‐ and two‐dimensional ^1^H and ^13^C NMR experiments and compared with the existing literature data [14].

### 4.2 Maintenance of Parasites and Mammalian Cells

*T. cruzi* (Y strain) was kindly provided by Professor Dr. Sergio de Albuquerque (Faculty of Pharmaceutical Sciences of Ribeirão Preto, University of São Paulo, Ribeirão Preto, Brazil) and maintained in vivo in male BALB/c mice (Taconic Biosciences, RRID:IMSR_TAC:BALB), obtained at the Central Animal Facility of the Ribeirão Preto, through routine blood passages at the peak of parasitemia (7 days post‐infection). Bloodstream trypomastigotes were collected via cardiac puncture and subsequently used to infect cell cultures in vitro. All experimental procedures were approved by the Animal Ethics Committee of the University of Franca (Approval number: 2061190421) and conducted in accordance with Brazilian regulations and institutional guidelines for good animal care practices.

C2C12 cells (*Mus musculus* myoblast cell line, RRID:CVCL_0188) were obtained from the Cell Bank of Rio de Janeiro (HUCFF, UFRJ, RJ, Brazil) and cultured in Dulbecco's Modified Eagle Medium (DMEM; Cultilab, Campinas, SP, Brazil, Cat# 460) supplemented with 5% heat‐inactivated fetal bovine serum (FBS; Cultilab, Campinas, Brazil, #Cat 63), penicillin (10 000 IU mL^−1^), and streptomycin (10 mg mL^−1^) (Sigma–Aldrich, Cat# 4333). Cultures were incubated at 37 °C in a humidified atmosphere containing 5% CO_2_ incubator (Sanyo, Osaka, Japan). For cell infection, C2C12 cells were exposed to bloodstream trypomastigotes at a ratio of 10 parasites per myoblast (10:1) in DMEM culture medium (Cultilab) and incubated following the standard operating procedures established in the laboratory for 7 days. Trypomastigotes were subsequently collected from the culture supernatant as described by Pagotti et al. (2021) [5].

### 4.3 In Vitro Trypanocidal Assay

For the experiment against the trypomastigote forms, the **SEBz** and **GUT/XAN, OBL**, and BNZ (LAFEPE—Pernambuco, BR) were previously dissolved in dimethyl sulfoxide (DMSO, Synth‐ Diadema, Brazil, Cat#  01D1011.01.BJ) and added to the wells of 96‐well plates (Kasvi—São José dos Pinhais, Brazil, Cat# K26‐096 V‐N) by serial dilution at concentrations ranging from 6.25 to 200 µg mL^−1^ for the extract and from 1.56 to 50 µM for the compounds. Next, the trypomastigote form was adjusted to a final concentration of 1 x 10^6^ parasites/well in supplemented DMEM medium (Cultilab) and added to each well. The plates were incubated at 37 °C in a humidified atmosphere containing 5% CO_2_ (Sanyo) for 24 and 48 h. After incubation, trypanocidal activity was evaluated by quantifying the number of viable (motile and morphologically intact) parasites using a Neubauer chamber (Kasvi, Cat# K5‐0011) under an optical microscope (Nikon, New York, USA) [5].

To evaluate the activity against the intracellular amastigote forms, C2C12 cells were seeded at a density of 5 × 10^5^ cells/mL in 24‐well plates (Kasvi, Cat# K12‐024), each containing a 13 mm round coverslip (Kasvi, Cat# K5‐0013), and incubated for 24 h at 37 °C in a humidified atmosphere with 5% CO_2_ (Sanyo). After incubation, non‐adherent cells were removed by washing with fresh culture medium, and the adherent cells were infected with bloodstream trypomastigotes at a ratio of 10 parasites per myoblast (10:1) for 48 h under the same incubation conditions [33]. Following infection, non‐internalized trypomastigotes were removed by washing the wells with fresh culture medium. **SEBz** and the compounds **GUT/XAN, OBL**, and BNZ (LAFEPE) were then added at concentrations previously determined to maintain at least 60% cell viability, as assessed by the 3‐(4,5‐dimethylthiazol‐2‐yl)‐2,5‐diphenyl tetrazolium bromide (MTT) assay. The extract and compounds were applied for 48 h at concentrations ranging from 0.78 to 12.5 µg mL^−1^ for the extract and 0.78 to 12.5 µM for the compounds, except for BNZ, which was tested at concentrations ranging from 1.56 to 50 µM. After the period, cells were washed with phosphate‐buffered saline (PBS, 1X), fixed with methanol (Synth, Cat# 162728247) for 5 min, and stained with Giemsa (Synth, Cat# 00G1003.08.AB) for 30 min. Coverslips were mounted on slides and examined under transmitted light microscopy (Nikon) at 100× magnification. The number of intracellular amastigotes was determined by randomly counting 200 cells per sample. Negative control groups (trypomastigote and intracellular amastigote forms) were maintained under the same conditions and cultured in DMEM containing 0.1% DMSO (Synth) [5].

### 4.4 Ultrastructural Damage Analysis

To assess ultrastructural changes in the parasites, TEM was employed. Trypomastigote forms (1 × 10^6^ cells/mL) were exposed to **SEBz** and the compounds **GUT/XAN, OBL**, and BNZ (LAFEPE) at their respective EC_50_ concentrations for 24 h. After incubation, the parasites were centrifuged and fixed in a solution of 3% glutaraldehyde (Sigma‐Aldrich, Cat# G7651) prepared in 0.1 M phosphate buffer (pH 7.2) for 24 h. Following fixation, the samples were washed twice with PBS and prepared for TEM, according to the methodology described by Candido et al. [49]. Microscopic analysis was performed using a JEOL JEM‐100CXII transmission electron microscope (JEOL). Parasites cultured in DMEM medium containing 0.1% DMSO served as the negative control.

### 4.5 Cytotoxic Activity

C2C12 myoblast cells were counted using a Neubauer chamber (Kasvi) and adjusted to a concentration of 2 × 10^5^ cells/well. Subsequently, they were seeded in a 96‐well culture plate with supplemented DMEM medium (Cultilab). The extract and compounds previously dissolved in DMSO (Synth) were added at concentrations ranging from 6.25 to 200 µg mL^−1^ for the extract and from 1.56 to 50 µM for the compounds and incubated at 37°C in the presence of 5% CO_2_ (Sanyo) for 24 and 48 h. Afterward, the cells were washed, and 20 µL of a solution containing 7 mg of MTT (Sigma–Aldrich, Cat# 475989) in PBS was added to each well. The plates were incubated at 37°C and in a 5% CO_2_ atmosphere for 4 h, and the formazan precipitate was solubilized with 100 µL of isopropyl alcohol (Sigma‐Aldrich, Cat# 563935). The absorbance was read at 550 nm with a spectrophotometer (Libra S12; Biochrom Corp, Miami, USA) [50]. Cells were incubated with DMEM containing 0.1% DMSO as the negative control, and with 25% DMSO as the positive control for cytotoxicity [51].

### 4.6 Hemolytic Activity

The hemolytic activity assay was conducted as described by Lazcano‐Pérez [52] with adaptations. Approximately 5 mL of a 3% suspension of defibrinated sheep erythrocytes (Laborclin‐Pinhais, Brazil, Cat# 520246) was prepared in 0.9% saline solution (Synth, Cat# 01C1060.01.AG). Approximately 50 µL of diluted erythrocytes was added to each well of a sterile 96‐well plate (Kasvi), and the samples previously diluted in DMSO were added at concentrations ranging from 6.25 to 200 µg mL^−1^ for the extracts and from 1.56 to 50 µM. The plates were then incubated at room temperature for 3 h, and the hemolysis was determined by hemoglobin release, quantified by the absorbance of the supernatants at 415 nm, read using a spectrophotometer (Libra S12 – Biochrom). The negative control consisted of erythrocytes in a 0.9% NaCl solution containing 0.1% DMSO, while the positive control consisted of erythrocytes in water.

### 4.7 Statistical Analysis

In vitro experiments were performed in triplicate and repeated three times. The EC_50_, CC_50_, and HC_50_ values were calculated as the mean obtained from each individual experiment using nonlinear regression of dose–response curves. The SI, which indicates parasite toxicity as compared to the host, was calculated as the ratio between CC_50_ and EC_50_. Data were statistically analyzed by two‐way analysis of variance followed by Dunnett's comparison. The statistical analyses were performed by using GraphPad Prism 5 (GraphPad Software, San Diego, California, USA, RRID:SCR_002798).

## Author Contributions

**Nicoli D. Oliveira**, **Mariana C. Pagotti**, **Lucas A. de L. Paula**, **Mariana Z. Paixão**, and **Daiane A. dos Santos** performed the biological assays, analyzed the data, and wrote the manuscript. **Rodrigo C. S. Veneziani** and **Renata Faleiros de Freitas** performed the phytochemical procedures. **Sérgio R. Ambrósio**, **Jairo K. Bastos**, and **Lizandra G. Magalhães** designed the experiments, project administration, and funding acquisition.

## Funding

This study was supported by the National Council for Scientific and Technological Development, Brazil–CNPq (Fellowships: 308903/2021‐8, 307778/2021‐5, 304425/2025‐7, 307707/2021‐0, and 304423/2025‐4) Coordination for the Improvement of Higher Education Personnel, Brazil‐CAPES (Finance code 001, process number 88887.605060/2021‐00) and São Paulo Research Foundation, Brazil‐FAPESP (2017/04138‐8 and 2018/05080‐6). The Article Processing Charge for the publication of this research was funded by the Coordenação de Aperfeiçoamento de Pessoal de Nível Superior ‐ Brasil (CAPES) (ROR identifier: 00x0ma614).

## Conflicts of Interest

The authors declare no conflicts of interest.

## Data Availability

The authors have nothing to report.
